# Correlation between Choroidal Vascularity Index and Outer Retina in Patients with Diabetic Retinopathy

**DOI:** 10.3390/jcm11133882

**Published:** 2022-07-04

**Authors:** Patryk Sidorczuk, Iwona Obuchowska, Joanna Konopinska, Diana A. Dmuchowska

**Affiliations:** Ophthalmology Department, Medical University of Bialystok, 24a M. Sklodowskiej-Curie, 15-276 Bialystok, Poland; iwonaobu@wp.pl (I.O.); joannakonopinska@o2.pl (J.K.)

**Keywords:** choroid, choroidal thickness, choroidal vascularity index, diabetic macular edema, OCT, outer retina, outer retinal layer, outer nuclear layer

## Abstract

The choroid supplies blood to the outer retina. We quantified outer retinal and choroidal parameters to understand better the pathogenesis of diabetic retinopathy (DR) and diabetic macular edema (DME). The retrospective cross-sectional single-center study included 210 eyes from 139 diabetic patients and 76 eyes from 52 healthy controls. Spectral-domain optical coherence tomography (OCT) was carried out with a Spectralis HRA + OCT imaging device. The outer retinal layer (ORL), outer nuclear layer (ONL), and choroidal thicknesses were assessed along with the choroidal vascularity index (CVI). The presence of DR, whether with DME or without, was associated with choroidal thinning (*p* < 0.001). Compared with the controls, patients with DR without DME presented with lower ORL and ONL thickness (*p* < 0.001), whereas those with DR and DME had higher values of both parameters (*p* < 0.001). Significant correlations between outer retinal and choroidal parameters were found only in patients with DR without DME (ORL with choroidal thickness: *p* = 0.003, rho = 0.34; ORL with CVI: *p* < 0.001, rho = 0.49, ONL with CVI: *p* < 0.027, rho = 0.25). No correlations between choroidal and outer retinal parameters were observed in the controls and patients with DR and concomitant DME. Aside from diabetic choroidopathy, other pathogenic mechanisms seem to predominate in the latter group.

## 1. Introduction

The macula is supplied with blood from two independent sources. The inner retina, located between the internal limiting membrane (ILM) and the outer plexiform layer (OPL), is perfused by the central retinal artery, whereas the outer retina, spreading between the outer border of the OPL and the Bruch’s membrane (BM), is supplied mainly by the choroid [1,2]. Since no retinal vasculature exists in the foveal region, compromised choroidal blood flow may lead to photoreceptor dysfunction [3,4,5,6]. Many previous studies demonstrated that in patients with diabetic macular edema (DME), the integrity of the ellipsoid zone and external limiting membrane (ELM) is closely associated with visual function [7,8,9,10]. Furthermore, the thickness of some outer retinal layers, e.g., total outer retina [11,12,13], photoreceptor outer segments [14,15], retinal pigment epithelium (RPE) [16,17], and retinal tissue between the plexiform layers [18], was shown to correlate with visual acuity. 

Diabetes may lead to choroidal abnormalities similar to those observed in the retina, such as microaneurysms, dilatation and obstruction of the vessels, vascular remodeling with increased vascular tortuosity, vascular dropout, focal vascular non-perfusion, atrophy of Sattler’s layer and Haller’s layer, and choroidal vascularization [19,20,21,22]. Choroidopathy may trigger the development of retinopathy due to retinal tissue hypoxia and overexpression of vascular endothelial growth factor (VEGF); this contributes to further retinal damage and DME [5,20]. However, it is still unclear whether choroidopathy precedes, accompanies, or follows the retinal changes [3,22,23,24]. Moreover, the exact contribution of diabetic choroidopathy to the diabetes-associated damage of the neuroretina and occurrence of DME remains poorly understood [22].

DME, the major cause of severe vision loss in patients with diabetes, can occur at any stage of diabetic retinopathy (DR) and affects both the outer and inner retina [25,26]. DME is defined as an abnormal increase in intra- and extracellular fluid volume in the macula. This multifactorial condition involves many complex mechanisms, including the breakdown of the inner- and outer blood-retinal barrier (BRB) [27,28]. Other underlying pathomechanisms of DME include ischemia, neurodegeneration, and edema [29].

Optical coherence tomography (OCT) is the primary tool to visualize the retina and choroid in healthy persons and diabetic patients with DME or without. To assess the relationship between the OCT-based characteristics of the choroid and outer retina, we divided the latter into the outer retinal layer (ORL) and outer nuclear layer (ONL), with the ELM as a border separating the two. ELM is a marker of photoreceptor integrity, and its disruption is associated with visual impairment in DME [7,8,9,10]. ELM is an intercellular junction between the Müller cells and photoreceptor cells, constituting a barrier for macromolecules [30]; a disruption of ELM may result in the migration of blood components into the outer retinal layers and resultant exacerbation of photoreceptor damage. ELM can be altered due to hyperglycemia [24]. ORL includes outer and inner segments of photoreceptors and RPE. The outer segments contain discs filled with opsin, responsible for absorbing photons for later signal transduction, whereas the inner segments are a reservoir of mitochondria needed for energy supply. Consequently, both inner and outer segments have an essential function in the visual pathway [11]. RPE constitutes the outer BRB. It removes the waste that remained after the phagocytosis of photoreceptors’ outer segments, provides nutrients for photoreceptors, absorbs light, pumps the fluid towards choriocapillaris, and controls retinal oxidative stress [24].

In the present study, we focused on choroidopathy, a component of DR and DME pathogenesis [3,5,20,22,23,24]. We aimed to explain the relationship between outer retinal and choroidal parameters. Aside from determining the choroidal thickness, we also calculated the choroidal vascularity index (CVI). CVI is a novel, OCT-based choroidal quantitative parameter providing more detailed information about the vascular component of the choroid across all its layers, i.e., choriocapillaris, Sattler’s layer, and Haller’s layer [31,32]. CVI has been proposed as a marker for early diagnosis, progression monitoring, and stratification of patients with various retinal and choroidal diseases and systemic conditions, including those of vascular or inflammatory origin [31]. Unlike the choroidal thickness, which depends on multiple physiological and pathological factors [31,32,33,34,35], CVI is considered a relatively stable parameter to evaluate changes in choroidal vasculature [36].

A number of previous studies analyzed either choroidal [6,17,34,37,38,39,40,41,42,43] or retinal characteristics [11,12,13,14,15,16,17,18,44] in diabetic patients with DR with concomitant DME or without. However, to the best of our knowledge, this is the first study to explore the link between the choroidal parameters (CVI and choroidal thickness) and the parameters of outer retinal thickness in such patients. A better insight into the pathogenesis of DR and DME may facilitate the stratification of patients in terms of prognosis and their qualification for novel treatments from the spectrum of personalized medicine.

## 2. Materials and Methods

The retrospective single-center cross-sectional study included 286 eyes from 191 patients (139 with DR and 52 controls). Medical records were analyzed for the period between 28 February 2017 and 20 February 2021. In 210 eyes from patients with diabetes, DR was confirmed by fluorescein angiography. The DR group was divided into two subgroups based on the presence of DME (DR + DME+) or lack thereof (DR + DME−). The control group (76 eyes) consisted of patients scheduled for routine ocular examination at the Department of Ophthalmology, University Teaching Hospital of Bialystok. 

All patients underwent spectral-domain OCT examination. DME was diagnosed whenever the retinal thickness in the central macular subfield (1 mm in diameter) of the Early Treatment Diabetic Retinopathy Study (ETDRS) grid sector was ≥300 µm and excluded if the thickness was <300 µm [45]. 

The exclusion criteria of the study were: prior posterior segment surgery or intravitreal injections, macular laser photocoagulation, ametropia ≥ 3.0 diopters, macular changes resulting from other ocular diseases, glaucoma, known ocular or systemic pathology potentially able to affect the choroidal vasculature, and insufficient quality of OCT images.

The study was conducted in line with the provisions of the Declaration of Helsinki and approved by the Ethics Committee at the Medical University of Bialystok (approval number APK.002.216.2020). Written informed consent was provided by all patients involved in the study.

### 2.1. Optical Coherence Tomography Images Acquisition and Analysis 

The protocol of the study was described elsewhere [46]. The OCT images were taken in mydriasis between 8 a.m. and 11 a.m. to avoid diurnal variation in the choroidal thickness. The images were independently assessed by two investigators (P.S. and D.A.D.) blinded to the clinical characteristics of examined eyes. 

**Figure 1:**
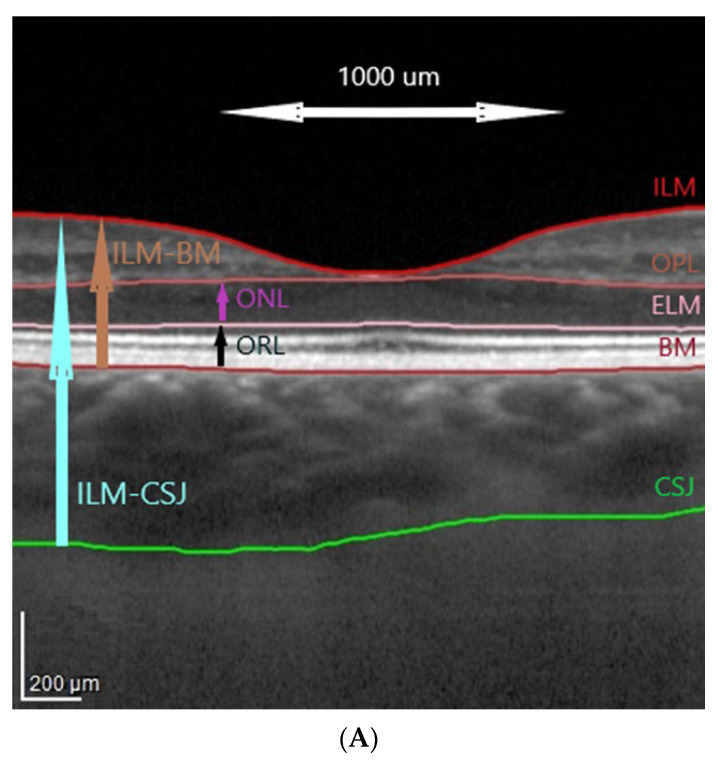

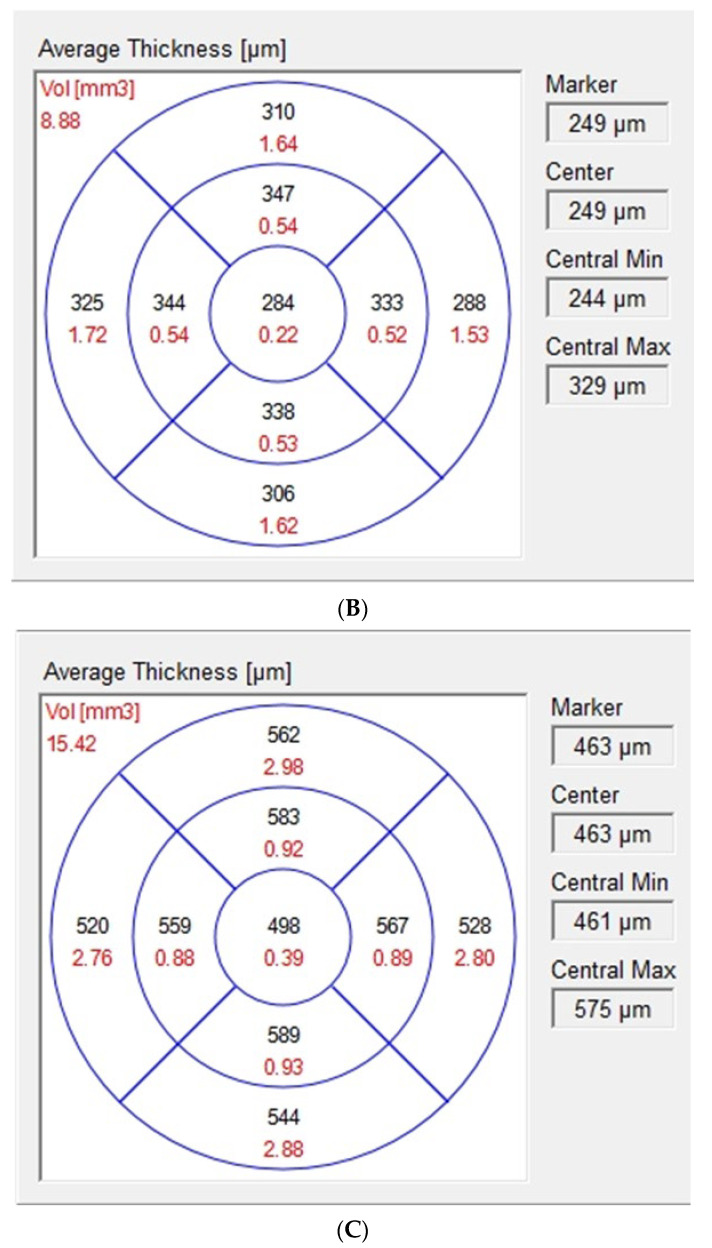
(**A**) Retinal layer segmentation and marking of the choroidal–scleral junction. (**B**) An ETDRS macular map showing retinal thickness (ILM-BM) and volume in 10 subfields. (**C**) An ETDRS macular map showing total thickness (ILM-CSJ) and volume in 10 subfields. Abbreviations: ILM, internal limiting membrane; OPL, outer plexiform layer; ELM, external limiting membrane; BM, Bruch’s membrane; CSJ, choroidal–scleral junction; ONL, outer nuclear layer; ORL, outer retinal layer; ETDRS, Early Treatment Diabetic Retinopathy Study.

Spectral-domain OCT was carried out with a Spectralis HRA + OCT imaging device with eye tracking (Heidelberg Engineering, Heidelberg, Germany). The protocol of the OCT imaging comprised of 25 horizontal raster scans (20 × 20°) and a linear B-scan centered at the fovea. The segmentation of the retinal layers was carried out automatically with the Spectralis software (version 6.7, Heidelberg Engineering, Heidelberg, Germany), as shown in Figure 1. The internal limiting membrane (ILM), outer plexiform layer (OPL), external limiting membrane (ELM), and Bruch’s membrane (BM) were detected automatically, and the choroidal–scleral junction was marked manually on each scan by shifting the BM line to the choroidal–scleral junction, as described previously [46]. Manual measurements were reviewed by the authors, and disagreements were resolved through discussion.

Based on ETDRS macular maps the values of choroidal parameters were obtained by subtracting retinal parameters (calculated automatically from the ILM to the BM, Figure 1A,B) from total parameters (calculated automatically from the ILM to the manually marked choroidal–scleral junction, Figure 1C). The fovea was checked and, if necessary, manually replotted. The outer retina was defined as the ONL and the ORL sum. The ONL and the ORL were defined according to the Heidelberg Spectralis HRA-OCT software, with the ONL as an area between the outer border of the OPL and the ELM and the outer retinal layer (ORL) as an area between the ELM and the BM (Figure 1A). The choroid was defined as an area between the BM and the choroidal–scleral junction. 

The values for the central 1 mm ring (within a 500 µm radius from the center of the macula) were extracted from the ETDRS macular map. Average central macular thickness at the ONL and ORL was calculated by the OCT software. Choroidal central macular thickness was calculated by subtraction, as described above.

### 2.2. Binarization of Subfoveal Choroidal Images

Binarization and segmentation of the images were done with ImageJ software (http://imagej.nih.gov/ij, accessed on 5 May 2021, version 1.49, U. S. National Institutes of Health, Bethesda, MD, USA), using the protocol proposed by Sonoda [32,47]. Briefly, the area within a 500 µm distance nasally and temporally from the fovea was analyzed on the horizontal scan across the fovea using the polygon selection tool, with the BM as the upper margin and the choroidal–scleral junction as the lower margin. Luminal area (LA) and total choroidal area (TCA) were measured. Stromal area (SA) was calculated, and CVI was determined as the LA to TCA ratio [31,32] (Supplementary Figure S1A–D). The inter-grader reliability was measured by the absolute agreement model of the intraclass correlation coefficient (ICC). ICC values for choroidal parameters were greater than 0.8, which indicated good agreement. With Bland–Altman plot analyses, the fixed and proportional bias were excluded.

### 2.3. Fluorescein Angiograms Acquisition and Analysis

Fluorescein angiography was performed with a Spectralis HRA + OCT imaging device (Heidelberg Engineering, Heidelberg, Germany) according to the standard procedure. The images were used to assess the severity of DR according to the ETDRS criteria [48,49].

### 2.4. Statistical Analysis

Statistical analyses were carried out with R software (version 3.5.1, R Foundation for Statistical Computing, Vienna, Austria). Descriptive statistics included numbers (percentages within each group) for nominal variables and means ± standard deviations (SD) for continuous variables. The normality of the distribution was verified with the Shapiro–Wilk test, on the basis of skewness and kurtosis values, as well as based on visual inspection of histograms. Between-group comparisons were carried out with a chi-square test or Fisher exact test for nominal variables and one-way ANOVA for continuous variables, whichever appropriate. Whenever the result of the ANOVA was statistically significant, Tukey post hoc test was applied. The relationships between pairs of continuous variables were verified based on Spearman’s correlation analysis. Linear univariate and multivariate regression analysis was carried out to assess relationship between choroidopathy and outer retinal thickness. Multivariate models were based on stepwise approach with AIC criterium, and independent variables with *p* < 0.157 in univariate analysis were included into multivariate models [50]. Both univariate and multivariate models were adjusted for covariates: age, sex, DR severity, and PRP. All tests were two-tailed with α = 0.05.

## 3. Results

### 3.1. Baseline Characteristics

The study included a total of 191 patients (286 eyes). Patients with DR with concomitant DME (DR + DME+) or without (DR + DME−) and the controls did not differ significantly in terms of age, sex, and spherical equivalent. Baseline characteristics of both groups of patients with DR and the controls are presented in Table 1.

### 3.2. Between-Group Comparison of Outer Retinal and Choroidal Parameters

Healthy controls presented with significantly higher choroidal thickness and CVI values than patients with DR (Table 2). However, the three groups did not differ significantly in terms of other choroidal parameters, i.e., luminal area (LA), stromal area (SA), and total choroidal area (TCA).

Compared with healthy controls, patients from the DR + DME− group presented with a lower thickness of both components of the outer retina, ORL and ONL. Meanwhile, the values of both these parameters in patients from the DR + DME+ group were significantly higher than in the control group.

### 3.3. Intra-Group Correlations and Regressions between Outer Retinal and Choroidal Parameters 

We analyzed correlations between the outer retinal (ONL and ORL) and choroidal parameters (choroidal thickness, CVI, LA, SA, and TCA) within each of the three groups (Table 3). No significant correlations between the choroidal and outer retinal parameters were found in the controls and DR + DME+ group. In DR + DME− group, however, ORL correlated positively with choroidal central macular thickness, CVI, and LA, and a positive correlation was found between ONL and CVI. 

Based on univariate regression analysis (Table 4), ORL central macular thickness (1-mm diameter) was associated with choroidal central macular thickness (β = 0.03, *p* = 0.019), CVI (β = 43.70, *p* < 0.001), LA (β = 18.73, *p* = 0.004), and TCA (β = 10.20, *p* = 0.021). Multivariate model for ORL indicated that CVI alone (β = 43.70, *p* < 0.001) was the best determinant of ORL with R^2^ = 0.26 (R^2^ adj. = 0.21). Inclusion of other parameters did not increase the quality of model. Low value of R^2^ indicated presence of other factors additional to this analysis impacting the thickness of ORL.

For ONL univariate regression analysis did not identify significant associations.

## 4. Discussion

As the demand of the outer retina for oxygen and nutrients is primarily covered by diffusion from the choroidal circulation [5], we decided to quantify outer retinal and choroidal parameters in patients with DR. Our study showed that patients with DR had the lower choroidal thickness and CVI than healthy controls. While the thickness of two components of the outer retina, ORL and ONL, in patients from the DR + DME− group was lower than in the controls, patients from the DR + DME+ group presented with higher values of these parameters than persons from the control group. Additionally, in patients from the DR + DME− group, ORL correlated positively with choroidal central macular thickness and LA; furthermore, both ORL and ONL correlated positively with CVI in this group of patients.

Choroidal thickness varies depending on multiple physiological and pathological factors, such as age, sex, refraction, axial length, time of the day, DME, DR severity and PRP, duration, and control of DM [31,32,33,34,35,40]. Our study groups did not differ significantly in terms of age, sex, spherical equivalent, DR severity, and PRP rate, and hence, a confounding effect of these variables was unlikely. CVI is a more independent measure [31,36] and therefore was considered a primary variable analyzed in this study.

In our study, healthy controls presented with significantly higher choroidal thickness than patients with DR, whether with concomitant DME or without. Although published data in this matter are inconclusive, our findings are in line with most previous studies [6,17,34,37,38,39]. Wang et al. observed increased choroidal thickness at the early stages of DR, followed by a decrease in this parameter with DR progression; meanwhile, DME was not significantly associated with choroidal thickness. Those findings suggest that alterations in choroidal parameters may play a role in the pathogenesis of DR [51]. In our study, the presence of DR was associated with a substantial decrease in CVI, with a statistically significant difference in this parameter observed between the DR + DME+ group and the controls; this observation is consistent with the results of previous studies [40,41,42,43]. However, we found no significant between-group differences in other choroidal parameters, LA, SA, and TCA. In our previous study, which centered around the choroid but not the outer retina, patients with various types of DME (cystoid, diffuse, with subretinal fluid) presented with lower CVI and choroidal thickness than the controls [46].

Both components of the outer retina, ORL and ONL, were thinner in the DR + DME− group and thicker in the DR + DME+ group when compared with healthy controls. Our findings are consistent with the results published by Sim et al., who reported outer retinal thinning in eyes with diabetic macular ischemia without macular edema and found outer retinal thickening in eyes with macular edema [12]. According to those authors, the ischemic thinning might be masked by coexisting edema. Our findings seem to support this hypothesis. We found significant differences in ORL and ONL thickness in patients with DR with concomitant DME and without. Thinning of the outer retina reflects neurodegeneration, which might be a reason behind the reduced outer retinal thickness in the DR + DME− group. Reduced choroidal thickness associated with DR may lead to the hypoxia of retinal tissues, resultant impairment of outer BRB, development of DME, and further progression thereof [6]. At a molecular level, hyperglycemia is associated with the activation of various pathways, including polyol pathway, protein kinase C pathway, generation of advanced glycation end-products, inflammation, and oxidative stress [24,52]. Activation of those pathways affects retinal and choroidal vessels and neurons, RPE cells, and glial cells [26,53].

Our findings are partly in agreement with the results published by Wang et al., according to whom DR patients presented with significantly lower choroidal, ORL, and RPE thickness than the controls, without significant differences observed between patients with concomitant DME and without [17]. Damian et al. found thinner ONL and ORL in patients in whom diabetes coexisted with mild DR or lack thereof, without concomitant DME [54]. Other authors reported outer retinal atrophic changes in DME [13] with PROS (photoreceptor outer segment) shortening [13,44].

In the present study, ORL thickness in the DR + DME− group correlated significantly with choroidal central macular thickness, CVI, and LA; a significant correlation between ONL and CVI was found in this group as well. Multivariate model for ORL indicated that CVI alone was the best determinant of ORL. To the best of our knowledge, none of the previous studies documented such correlations in patients with DR and concomitant DME. Damian et al. analyzed correlations between choroidal and outer retinal parameters in diabetic patients with mild DR or lack thereof, without concomitant DME. They found significant correlations between CVI and RPE thickness as well as between choroidal thickness and photoreceptor layer thickness [54]. While the results of that study could be partially consistent with our findings in the DR + DME− group, the analyzed cohort differed considerably, as it included patients with mild or absent DR.

Consistently with previous reports [54,55], we found no significant correlations between choroidal parameters (CVI and choroidal thickness) and outer retinal parameters in healthy controls.

Similarly, no significant correlations between retinal and choroidal parameters were observed in the DR + DME+ group. Gerendas et al. analyzed total retinal thickness and choroidal thickness in patients with DME and also did not find a significant correlation between these parameters [56]. However, in our present study, we considered outer retinal thickness rather than total retinal thickness, and hence, our findings should not necessarily be directly compared with those reported by Gerendas et al. [56] The lack of correlation between outer retinal parameters and choroidal parameters in patients with DME might reflect complex pathogenesis of this condition. All cells maintain internal homeostasis due to the existence of membrane transport systems that control the inflow and outflow of ions from the cell. Many pathological conditions (e.g., ischemia) are not only associated with the disruption of the BRB but may also lead to the damage of membrane ionic channels and resultant cellular swelling. Thus, neuronal and/or glial swelling may often be a component of retinal edema; probably, this refers in particular to the areas of ischemic capillary loss where severe metabolic insult occurs, and no viable capillaries survive to generate extracellular fluid [57]. Consequently, different stages of macular edema, i.e., cytotoxic (intracellular) versus vasogenic (extracellular), need to be considered and analyzed separately [58]. Moreover, patients may differ in terms of the disease pathways involved. Finally, ischemia, neurodegeneration, and edema can occur independently from one another [29,59,60].

In summary, we cautiously hypothesize that the blood supply is more than sufficient in healthy controls even with a variable flow through the choroid. However, this is not the case in patients with DR, as shown by decreased choroidal thickness and lower CVI values. The choroidal atrophy may lead to ischemia as a predominant pathogenetic mechanism of DR at this stage. Hypoxia may, in turn, cause RPE and photoreceptor degeneration, with resultant thinning of the outer retina. Further stages of the disease, when the disruption of the BRB occurs, and DME develops, involve multiple and complex pathogenetic mechanisms, and as a result, the relationship between choroidal circulation and outer retinal thickness is not that evident.

This study has some strengths, among them the inclusion of DME treatment-naïve patients and the analysis of age-, sex-, and refractive error-matched groups. All measurements were taken between 8 a.m. and 11 a.m. to avoid diurnal variations. The study included a homogenous group consisting solely of patients with spherical equivalent refractive error < 3.0 diopters. Compared with previous studies, we analyzed a larger number of patients with proliferative DR, often underrepresented in clinical research. Notably, we considered multiple choroidal parameters rather than only thickness. Further, this is the first published study to analyze a correlation between CVI and outer retinal thickness in such a group of patients.

We are well-aware of the potential limitations of this study. Due to the retrospective design of the study, the information about the type of diabetes, laboratory values, and the time of the onset of DME was unavailable. It also needs to be stressed that CVI was determined based on a 1 mm single foveal scan; this is a relatively common practice given that CVI is similar across all the ETDRS subfields [35]. Future advances in automated three-dimensional CVI evaluation may address this limitation. Furthermore, we focused solely on anatomical parameters; meanwhile, the analysis of correlations between functional parameters seems to be an interesting direction for future research. Additionally, we analyzed only a single mechanism involved in DR/DME development, i.e., choroidopathy, whereas the pathogenesis of these conditions is complex. We did not consider a recently discovered role of previously underappreciated, deep capillary plexus in satisfying the metabolic requirements of the outer retina [2]. Future studies may address this limitation by introducing OCT angiography in the study design. Unfortunately, the analysis of OCT angiographic images in patients with DME still constitutes a problem nowadays.

## 5. Conclusions

In conclusion, the presence of DR with concomitant DME or without is associated with changes in choroidal and outer retinal parameters. However, a significant correlation between outer retinal parameters and choroidal parameters was found only in the DR + DME− group, and no link between these parameters was observed in healthy controls and patients from the DR + DME+ group.

## Figures and Tables

**Table 1:** Baseline characteristics of patients with DR with concomitant DME or without and healthy controls.

	Overall	Group			*p*
		DR + DME+	DR + DME−	Controls	
Number of patients	191	49	90	52	
Number of eyes	286	76	134	76	
Age, years, mean ± SD	59.24 ± 14.43	61.50 ± 11.92	60.03 ± 13.19	55.73 ± 17.87	0.103
Sex, female, *n* (%)	100 (52.4)	25 (51.0)	47 (52.2)	28 (53.8)	0.960
Spherical equivalent, mean ± SD	0.32 ± 1.10	0.43 ± 1.07	0.23 ± 0.99	0.39 ± 1.28	0.371
DR severity, *n* (%)					
NPDR	135 (47.2)	50 (65.8)	85 (63.4)	-	0.766 ^1^
PDR	75 (26.2)	26 (34.2)	49 (36.6)	-	
PRP, *n* (%)					
No	138 (48.3)	48 (63.2)	90 (67.2)	-	0.650 ^1^
Yes	72 (25.2)	28 (36.8)	44 (32.8)	-	

Notes: Groups compared with chi-square test or Fisher exact test ^1^ for nominal variables and with ANOVA for continuous variables. DME defined as present (DME+) when central macular subfield retinal thickness ≥ 300 µm and absent (DME−) when central macular subfield retinal thickness < 300 µm. Abbreviations: DME, diabetic macular edema; DR, diabetic retinopathy; NPDR, non-proliferative diabetic retinopathy; PDR, proliferative diabetic retinopathy; PRP, panretinal photocoagulation.

**Table 2:** Choroidal and outer retinal parameters in patients with DR with concomitant DME or without and healthy controls.

	DR + DME+	DR + DME−	Controls	*p*	Post Hoc		
					DR + DME+ vs. DR + DME−	DR + DME− vs. Controls	DR + DME+ vs. Controls
Choroidal parameters							
CVI	0.62 ± 0.05	0.63 ± 0.05	0.65 ± 0.05	0.001	0.576	0.089	**<0.001**
LA (mm^2^)	0.24 ± 0.05	0.25 ± 0.08	0.25 ± 0.06	0.266			
SCA (mm^2^)	0.15 ± 0.03	0.14 ± 0.04	0.14 ± 0.03	0.204			
TCA (mm^2^)	0.39 ± 0.08	0.40 ± 0.01	0.39 ± 0.08	0.773			
Choroid (µm)	250.75 ± 45.61	265.34 ± 60.91	303.64 ± 72.77	<0.001	0.218	**<0.001**	**<0.001**
Central macular thickness (1 mm diameter)							
ORL (µm)	92.58 ± 24.10	84.20 ± 4.97	87.64 ± 3.52	<0.001	**<0.001**	0.157	0.052
ONL (µm)	141.70 ± 85.70	82.38 ± 18.03	92.13 ± 10.81	<0.001	**<0.001**	0.306	**<0.001**

Notes: Data presented as mean ± SD. Groups compared with ANOVA. In cases of statistically significant differences, Tukey post hoc test was applied. Significant *p* values in bold. Abbreviations: DME, diabetic macular edema; DR, diabetic retinopathy; CVI, choroidal vascularity index; LA, luminal area; SA, stromal area; TCA, total choroidal area; ORL, outer retinal layer; ONL, outer nuclear layer.

**Table 3:** Correlations between outer retinal and choroidal parameters in patients with DR with concomitant DME or without and healthy controls.

Correlation:	DR + DME+		DR + DME−		Controls	
	Rho	*p*	Rho	*p*	Rho	*p*
ORL central macular thickness (1 mm diameter)						
Choroidal central macular thickness (µm)	0.23	0.160	**0.34**	**0.003**	0.27	0.069
CVI	−0.01	0.953	**0.49**	**<0.001**	0.12	0.426
LA (mm^2^)	0.23	0.171	**0.41**	**<0.001**	0.19	0.191
SA (mm^2^)	0.17	0.314	0.12	0.293	0.05	0.719
TCA (mm^2^)	0.18	0.285	0.32	0.005	0.17	0.242
ONL central macular thickness (1 mm diameter)						
Choroidal central macular thickness (µm)	−0.07	0.683	0.01	0.932	−0.27	0.058
CVI	0.35	0.031	**0.25**	**0.027**	0.14	0.336
LA (mm^2^)	0.08	0.641	0.09	0.430	−0.02	0.871
SA (mm^2^)	−0.22	0.191	−0.20	0.077	−0.16	0.289
TCA (mm^2^)	−0.03	0.856	−0.01	0.981	−0.09	0.558

Notes: rho, Spearman’s correlation coefficient. Only the results for a single eye from each patient were considered during the analysis, *n* = 191. Since the dataset included the results for a single eye from each patient, there was no violation of the independence assumption between observations for correlation analysis. Significant *p* values with rho values in bold. Abbreviations: DME, diabetic macular edema; DR, diabetic retinopathy; CVI, choroidal vascularity index; LA, luminal area; SA, stromal area; TCA, total choroidal area; ORL, outer retinal layer; ONL, outer nuclear layer.

**Table 4:** Univariate linear regression for ORL and ONL in patients with DR without concomitant DME.

	ORL Central Macular Thickness (1 mm Diameter)				ONL Central Macular Thickness (1 mm Diameter)			
	β	SE	*p*	R^2^/R^2^ adj.	β	SE	*p*	R^2^/R^2^ adj.
Choroidal central macular thickness (µm)	0.03	0.01	**0.019**	0.14/0.08	0.03	0.04	0.395	0.23/0.18
CVI	43.70	9.93	**<0.001**	0.26/0.21	67.03	36.32	0.069	0.26/0.21
LA (mm^2^)	18.73	6.22	**0.004**	0.17/0.11	7.94	21.92	0.718	0.23/0.17
SA (mm^2^)	12.29	12.54	0.330		−34.52	41.74	0.411	0.23/0.18
TCA (mm^2^)	10.20	4.32	**0.021**	0.13/0.07	−0.70	14.91	0.963	0.23/0.17

Notes: β, beta estimate; SE, standard error; R^2^ adj., adjusted R-squared. Only the results for a single eye from each patient were considered during the analysis, *n* = 90. All models were adjusted for covariates: age, sex, DR severity, and PRP. Significant *p* vales in bold. Abbreviations: DME, diabetic macular edema; DR, diabetic retinopathy; CVI, choroidal vascularity index; LA, luminal area; SA, stromal area; TCA, total choroidal area; ORL, outer retinal layer; ONL, outer nuclear layer.

## Data Availability

All the materials and information will be available upon an e-mail request to the corresponding author. Names and exact data of the participants of the study may not be available owing to patient confidentiality and privacy policy.

## Supplementary Materials

The following supporting information can be downloaded at: https://www.mdpi.com/article/10.3390/jcm11133882/s1, Figure S1. Image binarization of the choroid and calculation of choroidal vascularity index (CVI).
