# Supplementary material for: Correlation between Choroidal Vascularity Index and Outer Retina in Patients with Diabetic Retinopathy
Source: J Clin Med. 2022 Jul 4;11(13):3882. doi: 10.3390/jcm11133882 (PMC9267134; doi:10.3390/jcm11133882)
Supplement: Supplementary file 1 [file jcm-11-03882-s001.zip › jcm-1778463-supplementary.pdf]

Supporting information to **"Correlation between choroidal vascularity index and outer retina in patients with diabetic retinopathy"**

Patryk Sidorczuk[1*], Iwona Obuchowska[1], Joanna Konopinska[1] and Diana A. Dmuchowska[1*]

[1] Ophthalmology Department, Medical University of Bialystok, 24a M. Sklodowskiej-Curie, Bialystok, 15-276, Poland; iwonaobu@wp.pl (I.O.); joannakonopinska@o2.pl (J.K.)

* Correspondence: patryk.sidorczuk@gmail.com (P.S.); diana.dmuchowska@umb.edu.pl (D.A.D.)

**Supplementary Figure S1.** Image binarization of the choroid and calculation of choroidal vascularity index (CVI).

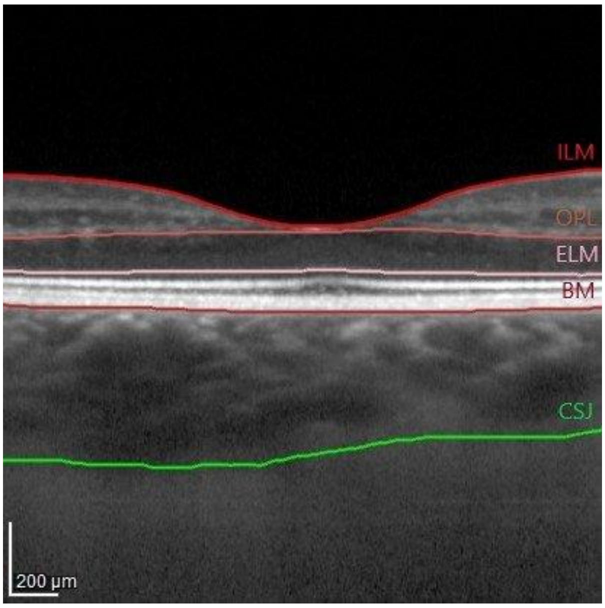


(A) The examined area was determined by Bruch's Membrane (BM, purple line) and choroidoscleral junction (CSJ, green line).

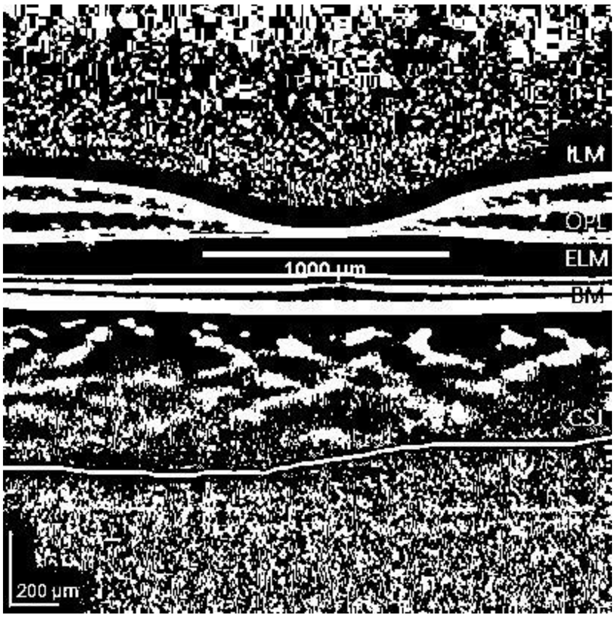


(B) The horizontal macular 1x1µm scan was uploaded in ImageJ software (http://imagej.nih.gov/ij, accessed on May 5, 2021, version1.49) and the image scale was set. Then, the image was converted to 8-bit. A line tool was used to draw the 1000µm line to determine the area beneath the fovea. The image was adjusted by the Niblack auto local threshold tool. The light pixels represented the stromal area (SA) and the black pixels and the dark pixels represented the luminal area (LA).

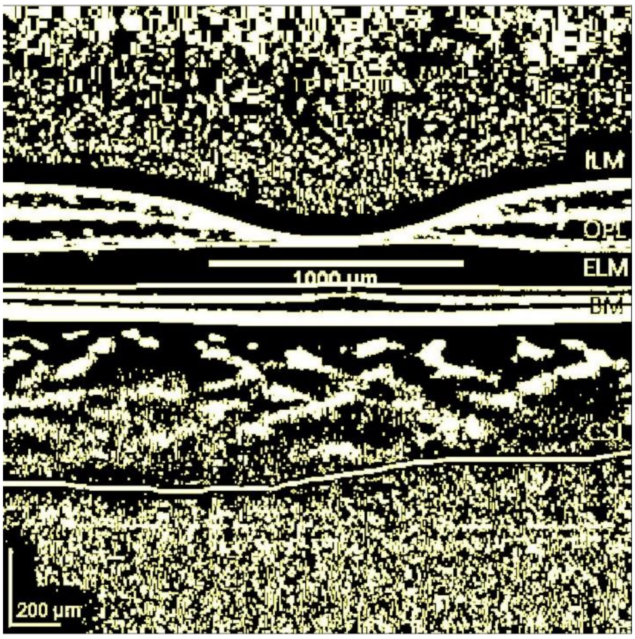


(C) The binarized image was reconverted to an RGB (red, green, blue) image, and the LA was highlighted using the threshold tool.

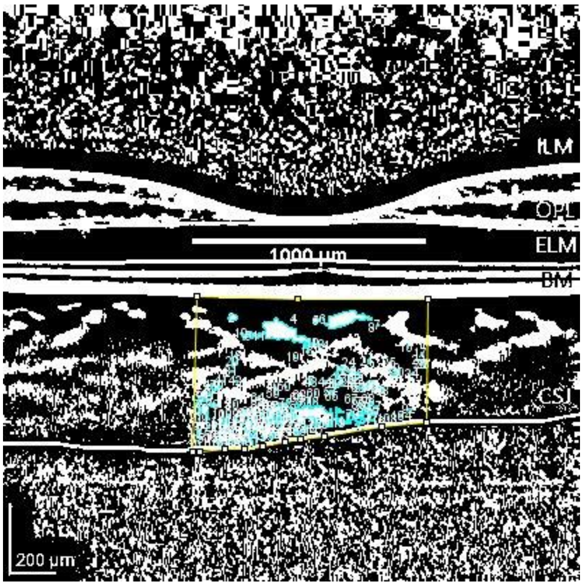


(D) The polygon selection tool was used to mark the 1000µm wide subfoveal region of interest (ROI) within the choroid (from BM to CSJ) termed the total choroidal area (TCA). It was selected and added to the ROI manager. The LA was automatically calculated by the ImageJ software. The stromal area (SA) was obtained by subtracting the luminal area (LA) from the TCA. The choroidal vascularity index (CVI) was calculated as the proportion of LA to TCA.
